# Characterization of Chronic Mechanical Irritation in Oral Cancer

**DOI:** 10.1155/2017/6784526

**Published:** 2017-04-06

**Authors:** Jerónimo P. Lazos, Eduardo D. Piemonte, Hector Eduardo Lanfranchi, Mabel N. Brunotto

**Affiliations:** ^1^Oral Medicine Department, Dentistry College, Universidad Nacional de Córdoba, Av. Haya de la Torre s/n, Ciudad Universitaria 5000, Córdoba, Argentina; ^2^Oral Medicine Department, Dentistry College, Universidad de Buenos Aires, Buenos Aires, Argentina; ^3^Oral Biology Department, Dentistry College, Universidad Nacional de Córdoba, Av. Haya de la Torre s/n, Ciudad Universitaria, Córdoba, Argentina

## Abstract

*Objective*. Oral mucosa could host many lesions originated by chronic mechanical irritation (CMI) from teeth or dentures, and it has been proposed as risk factor for oral cancer. Nevertheless, the features of CMI factors in oral cancer and other lesions are not assessed. The aim of this study is to describe CMI features regarding type (dental, prosthetic, and/or functional), localization, and time span.* Materials and Methods*. Three groups were studied in this cross-sectional study: Oral Cancer (OC); Chronic Traumatic Ulcer (CTU); and Benign Irritative Mechanical Lesions (BIML). All sources of mechanical irritation were included: dental, prosthetic, and functional.* Results*. 285 patients (176 females, 109 males) were studied: OC = 38, CTU = 44, and BIML = 203. The most frequent CMI factor was dental, followed by functional and prosthetic in all groups; 76.5% (*n* = 218) presented functional factors. Buccal mucosa (45%) and tongue (42%) were the most affected sites. Time of action of CMI displayed statistically significant differences between BIML, CTU, and OC groups, with a mean of 21, 33, and 49 months, respectively.* Conclusions*. CMI should be properly recorded with as much detail as alcohol and tobacco consumption. CMI associated lesions are produced by dental or prosthetic factors, usually in relation to functional factors, involving mainly tongue and buccal mucosa.

## 1. Introduction

Oral mucosa could host many lesions originated by chronic mechanical irritation (CMI) either from teeth or dentures. The most common CMI lesions are tongue/cheek biting* (morsicatio buccarum)*, frictional keratosis, indentations, chronic traumatic ulcer, papillary hyperplasia, denture-induced fibrous hyperplasia, and focal fibrous hyperplasia [1]. In addition, CMI could worsen preexisting oral lesions, such as bullous pathologies, oral lichen planus, leukoplakia, or aphthous stomatitis [2].

CMI is produced through low-intensity, sustained, and repeated action of an oral deleterious agent. The mechanic damage can be caused by teeth, dentures, and functional alterations, either through separate or combined action [3]. There are three types of CMI factors:* dental* (malpositions, sharp/broken teeth, and/or rough or defective restorations);* prosthetic* (ill-fitting dentures, rough/sharp/overextended flanges, and lack of retention/stability); and* functional* (swallowing, occlusal, and other dysfunctional disorders) [4].

CMI has been proposed as risk factor for oral cancer [4, 5]. Defective teeth and ill-fitting dentures have been mentioned in relation to oral cancer [6, 7]. This association is also supported by experimental studies of chemically induced carcinogenesis with CMI that showed an increase in cancer occurrence, higher malignancy grade, and a shorter latency period [8]. CMI per se may not be able to produce genetic mutations but may prompt epigenetic changes that ultimately inhibits DNA reparation and apoptosis [9]. This suggests that CMI could at least play a role as promoter and progressor in oral carcinogenesis. Thus, if a cancer has eventually started from another cause, CMI will probably hasten the process [10].

Chronic Traumatic Ulcer (CTU) is a relevant clinical finding because it represents the effect of a low-intensity and persistent CMI [11]. The cause (mechanical agent) and effect (lesion) relationship is usually clear [12]. It often appears in regions that are easily injured by teeth or dentures, such as lip, tongue, and buccal mucosa. In denture wearers it may also be found on floor of the mouth and on the mucobuccal fold. CTU usually exhibit a yellow base, whitish elevated margins, and a roughly oval shape that resembles the causative agent. Induration, often associated with CTU borders, is due to scar formation and chronic inflammatory cell infiltration. Initial lesions usually show variable degrees of pain, yet most chronic reactive ulcers may be painless, which is why CTU could have a lengthy evolution period. The histopathology displays an hyperplastic epithelium on the margins and a mixed granulation tissue throughout base and depth connective scarring [1, 3]. CTU has been linked to malignant transformation mainly through case reports [3, 13, 14] but several authors reject this possibility. Recurrent oral ulcerations have shown an increase of the risk of oral squamous cell carcinoma in nonsmokers and nondrinking individuals [15]. Moreover, CTU may clinically resemble a squamous cell carcinoma, so it would be proper to analyze it apart from other oral CMI lesions. Previous studies have shown a clear link between persistent inflammation and cancer, through the overexpression of genes regulating proliferation, angiogenesis, and immune evasion [16]. So, CMI could also play a role promoting a continuous inflammatory state.

It is noteworthy that CMI is an underregistered condition, and data of bad oral health (e.g., loss of teeth) does not necessarily reflect factors able to induce mucosal mechanical injuries. Also, it is not known if different sources of oral CMI (dental, prosthetic, and functional) could induce or change lesions differently. Furthermore, epidemiological studies of oral cancer and CMI do not include all the potentially mechanical factors described, nor relevant features such as localization and time length. Besides, CMI factors in oral cancer and Chronic Traumatic Ulcer (CTU) are not described. Therefore, the aim of the present study is to describe CMI features regarding type (dental, prosthetic, and/or functional), localization, and time span of the deleterious agent and, also, to analyze differences of CMI in oral cancer, CTU, and other irritiative mechanical lesions. The present study was not meant to find relationship between CMI and oral cancer and is complementary of a previously published paper [4].

## 2. Materials and Methods

This cross-sectional study was approved by the Research and Ethics Committee of the Córdoba Health Ministry, in accordance with the declarations of Nüremberg, Helsinki, and Tokyo of the World Medical Association. Informed consent was obtained from all individuals included in the study. The sample were patients seeking treatment in the Oral Medicine Department of Dentistry College, Universidad Nacional de Córdoba, from 2006 to 2010.

A* CMI case* was considered if oral mucosal lesions in relation to mechanical factors were presented, using Piemonte et al. criteria [4]. Three groups were established: Oral Cancer (OC); Chronic Traumatic Ulcer (CTU); and Benign Irritative Mechanical Lesions (BIML), in which other chronic mechanical lesions were included.

CMI was considered to be present, both on clinically healthy mucosa and worsening a previously diagnosed pathology, when all of the following conditions were registered:

1. Objective clinical lesion compatible with mechanical origin (e.g., erythema, atrophy, ulceration, keratosis, and hyperplasia) with evolution of over a month.
2. Mechanical factor present* before* the onset and/or modification of the lesions. This has been established through anamnesis.
3. Mechanical agent must be in direct contact with the lesion, during functional/parafunctional movements or decubitus position.

All sources of mechanical damage were included: dental, prosthetic, and functional. The last one includes both* dysfunctional*, which is an alteration of physiological function, for example, a swallowing disorder, and* parafunctional*, referring to habits that are different from physiological traits such as mastication, communication, swallowing, or breathing [17]. A common example of this cluster would be tongue interposition [18].

The OC group included oral squamous cell carcinoma and/or verrucous carcinoma (ICD-10 C00-C06) confirmed by biopsy. CTU was considered present when all the following requisites were met: (1) it should be fit with a mechanical lesion according to the aforementioned CMI criteria; (2) a yellow-base ulcer, whitish elevated margins; (3) painful or not; (4) healing takes place within 3-4 weeks after removing the mechanical causative agent. If a case had not healed after that period, a biopsy was performed in order to verify diagnosis. The BIML group were oral lesions associated with CMI* excluding* OC, CTU, and alveolar ridge keratosis, for example, tongue/cheek biting, frictional keratosis (outside alveolar ridges), indentations, papillary hyperplasia, denture-induced fibrous hyperplasia, focal fibrous hyperplasia, and denture stomatitis. Also, within the BIML group were included CMI aggravated lesions, such as leukoplakia, oral lichen planus, cheilitis, and recurrent aphthous stomatitis.

**Figure 1:**
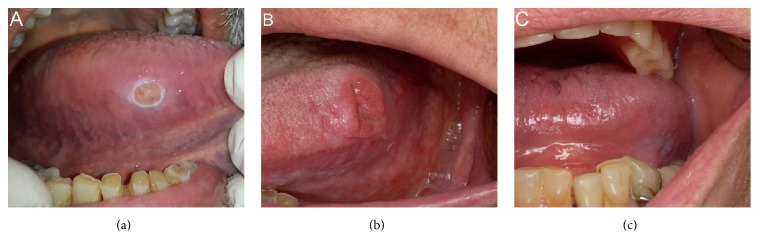
(a) Chronic Traumatic Ulcer (CTU) in association with sharp teeth and dysfunctional swallowing; (b) squamous cell carcinoma of tongue at first consultation; (c) same patient as (b); here a removable denture without retention (prosthetic factor) held in place by the tongue (functional factor) could be seen.

Clinical data were registered in a specific clinical form (sociocultural, genetic, environmental, anthropometric, medical, and dental). Oral cavity inspection was performed by previously calibrated dentists, through visual inspection and palpation of oral mucosa, teeth, and prosthetics devices (removable/fixed). Dysfunctional and parafunctional habits were also registered (Figure 1).

### 2.1. Statistical Model

Quantitative data were described using median values, and qualitative data were expressed as percentages. Data were processed using the Kruskall-Wallis nonparametric ANOVA test for unpaired samples, Chi-Square test, and Student's *t*-test. A *p* < 0.05 was set for significant differences. All statistical analyses were performed with Infostat (v. 2015, http://www.infostat.com.ar).

## 3. Results

A total of 687 individuals were registered, of which 285 (176 females, 109 males) who met the inclusion criteria were studied: OC = 38, 44 = CTU, and 203 = BIML, with a mean age of 45.7 years. Age and gender presented statistically significative differences, whereas there were no differences regarding tobacco and alcohol consumption between groups (Table 1).

**Figure 2:**
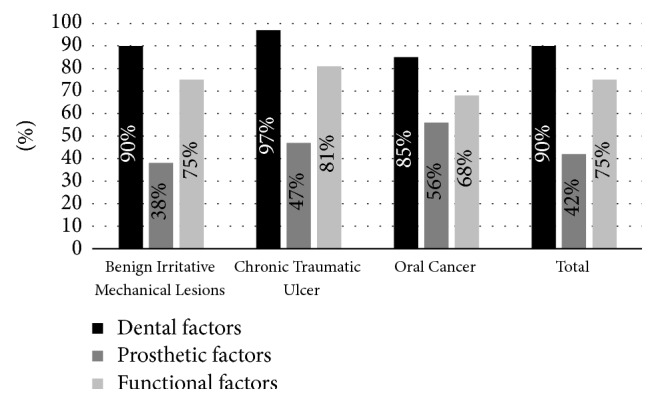
Distribution of chronic mechanical irritation factors.

The most frequent CMI factor was dental, followed by functional and prosthetic in all groups (Figure 2). Dental CMI presented no statistically significant differences in OC, UTC, and BIML (*p* = 0.26). Likewise, prosthetic and functional CMI were similar between groups (*p* = 0.09 and *p* = 0.37).

Dental Factors (DF) found were dental malposition, diastema, sharp/rough teeth, and/or restorations; sharp teeth were particularly related to CTU. The prosthetic factors (PF) found were sharp/rough dentures, denture retainers, overextended flanges, lack of retention, and/or stability. Among PF, denture use itself was not associated with any group. However, defective denture was mainly in relation to CTU and OC. Functional Factors (FF) included tongue interposition, sucking, biting, and others (e.g., tics and parafunctional habits). Only* tongue interposition* was associated with CTU and OC (Table 2).

Regarding CMI location, 35% presented lesions in multiple sites. Buccal mucosa (45%) and tongue (42%) were the most affected sites. Nevertheless, occurrence was different in each group. In BIML group, buccal mucosa (48%) and tongue (31%) were the most affected locations. Meanwhile, both in CTU and OC groups, tongue (CTU = 70%, OC = 68%) and buccal mucosa (CTU = 32%, OC = 45%) were the most injured sites. The rest of the mechanical lesions on other localizations presented in much lower percentages (hard and soft palate, lip, alveolar ridge, alveolar mucosa, and floor of the mouth).

In BIML group, 63% (*n* = 139) of the lesions were originated by CMI, while 37% (*n* = 81) were aggravated by CMI. Among lesions originated by CMI, we found denture stomatitis (29%, *n* = 41), tongue or cheek biting (28%, *n* = 40), focal fibrous hyperplasia (14%, *n* = 20), denture-induced fibrous hyperplasia (13%, *n* = 18). CMI aggravated lesions were leukoplakia (36%, *n* = 29), cheilitis (21%, *n* = 17), oral lichen planus (17%, *n* = 14) and recurrent aphthous stomatitis (13%, *n* = 11).

**Figure 3:**
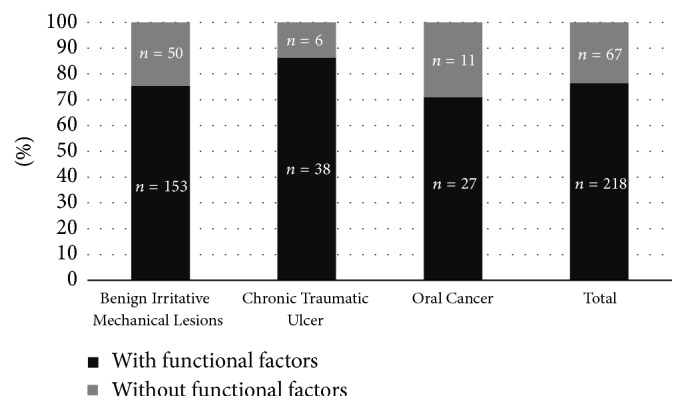
Distribution of functional chronic mechanical irritation factors.

Only 10% (*n* = 29) of the population had only one CMI factor (dental, prosthetic, or functional), whereas the rest had a combination of them. To such an extent that 76.5% (*n* = 218) presented functional factors, combined or not with dental and/or prosthetic factors, whereas 23.5% (*n* = 67) did not have functional factors (Figure 3). Chi-square analysis found no differences regarding functional factors in BIML, OC, and CTU groups (*p* = 0.207).

**Figure 4:**
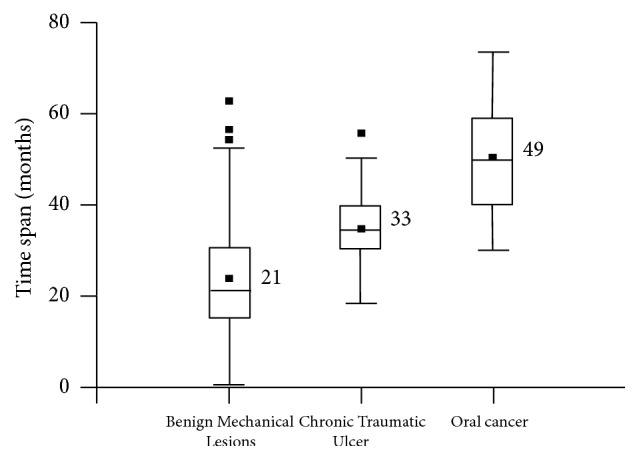
Time span for CMI of Benign Irritative Mechanical Lesions (BIML), Chronic Traumatic Ulcer (CTU), and Oral Cancer (OC).

The time of action of CMI displayed statistically significant differences between BIML, CTU, and OC groups (Kruskall-Wallis, *p* value < 0.0001), with a mean of 21, 33, and 49 months, respectively (Figure 4).

Assessment of time span showed that PF had a longer duration than dental factors in all groups, with a statistically significant difference: without FF: PF = 39.8 and DF = 27, *p* = 0.0001; with FF, PF = 41.28 and DF = 22.08, *p* < 0.0001 (Student's *t*-test; all values are expressed in months).

## 4. Discussion

In order to properly understand the findings of this study, we believe it is convenient to consider briefly some features of the population under study. OC cases presented a higher age than BIML and CTU groups, which is in agreement with previous papers, emphasizing the concept of cumulative damage along time [19].

Concerning gender, BIML and CTU groups had more females, in contrast with a slight prevalence of males in OC group. This could be because women usually seek professional attention more often than men, besides the historical predominance of males in oral cancer [20]. Tobacco and alcohol consumption were similar between all groups (BIML, CTU, and OC), which suggests that those factors might not have a confounding effect on the CMI attributed consequences.

In reference to CMI localization, it is not surprising that the most affected sites were tongue and buccal mucosa, considering that they are often in continuous contact with teeth and dentures. It is remarkable that CTU and OC cases exhibited similar locations (lateral border of the tongue), which was different for BIML group.

Even though most mechanical lesions of BIML group were originated by CMI, a significant percentage had lesions aggravated by CMI (35%, *n* = 71). Among them, we found potentially malignant disorders such as leukoplakia and oral lichen planus. This emphasizes that CMI could alter potentially malignant disorders of oral mucosa, providing an extra inflammatory background.

When the occurrence of mechanical factors (dental, prosthetic, and functional) was assessed, CMI origin was similar in BIML, CTU, and OC groups. This suggests that CMI lesions are not exclusively related to the mechanical factor but also to intrinsic individual traits or are dependent on not easily measurable features of CMI, like strength or regularity. However, certain CMI lesions showed statistical association with specific groups. Within dental factors, sharp teeth were related to CTU, which is in accordance with the results of Rosenquist et al., who found an increased risk of oral cancer with defective teeth [7].

As stated before, denture use itself was not associated with any group; yet defective dentures were in relation to CTU and OC. Many studies failed to find a statistical association between removable dentures and OC [21, 22]. They only assessed denture existence, but its condition was not recorded (lack of stability and/or retention, roughness, etc.). Therefore, they did not distinguish between dentures that could potentially injure oral mucosa—CMI cause—from the ones that would not. In the few studies that did register denture condition, defective dentures were statistically associated with higher oral cancer risk, which is in agreement with our findings [6, 7, 23–25].

Within functional factors, tongue interposition was strongly associated with CTU and OC. In those cases, the tip or borders of the tongue are trapped between dental arches approximately three times per minute in a normal swallowing pattern [26]. Consequently, when this practice is present for months or even years, the tongue is exposed to a continuous mechanical irritation. This emphasizes CMI as a persistent, repetitive condition, sustained over time, rather than an isolated unique event.

It is remarkable that functional factors were identified in more than two-thirds of the studied population, essentially combined with dental or prosthetic factors, a finding which has not been previously described. The FF presented themselves isolated to a very limited extent, and only 23.5% (*n* = 67) of the sample showed dental and/or prosthetic factors without FF. Therefore, this could explain why the sole existence of a defective tooth or denture was not deemed as a risk factor for OC in previous studies [27].

Many patients have defective teeth or dentures; however, despite having a potential source of CMI, few develop mechanical lesions. Thus, FF could facilitate oral mucosa invasion towards teeth, magnifying contact intensity. In addition, FF could also increase contact frequency, enabling the persistence of the mechanical injury. This emphasizes the need for identifying dysfunctions and parafunctions in order to properly detect oral CMI. Regardless, it should be stressed that CMI effect is an* objective clinical sign*, and could only be regarded as existing using the aforementioned criteria mentioned.

Furthermore, within FF, tongue interposition was in relation to CTU and OC, which may explain why the tongue is the most injured site in the oral cavity. This could also explain why the border of the tongue is the most frequent oral site for cancer [28], except for countries with particular habits (e.g., betel nut consumption) [29].

Another oral CMI feature was time span, meaning the time length of the injuring condition. This is mainly because it is unlikely to determine accurately the evolution time of CMI lesions, since many of them are asymptomatic. It is possible, however, to find how long has it been since teeth loss, fracture or malposition, or a denture malfunction, even with the patient memory bias. Time of CMI was not registered in previous studies of oral cancer.

Duration of CMI was longer in OC and CTU, with statistically significant differences with BIML group. Whereas in BIML group the average time span was 22 months, in CTU it was 33 months and in OC it was 49 months. Thus, CTU has a time progression which resembles closely CMI associated with OC. Similarly, CMI related to OC appears to be a long-lasting condition that covers a prolonged time span. Also, this discredits the notion that one or several accidental injuries could be related to OC. Instead, a prolonged, sustained, and repeated irritation is needed in order to create proper local conditions to foster carcinogenesis. CMI itself may not be able to cause oral cancer, but since OC is a multistage and multifactorial process, CMI could at least play a role. Consequently, CMI could foster oral carcinogenesis through various ways (e.g., enabling the absorption of chemical carcinogens and increasing cell proliferation), mainly because it allows a persistent inflammatory state. Thus, CMI could cooperate with other carcinogenic factors, which was demonstrated in animal experiments [8].

Regarding CMI duration according to the mechanical factor (dental or prosthetic), DF displayed a longer time span than PF. This could be because DF are not modifiable by the patient, thus acting uninterruptedly, and maybe with more strength. In contrast, the use of removable dentures (PF) could be easily discontinued. This could drastically reduce PF effective contact time on oral mucosa.

A fairly common oral lesion of CMI,* tongue or cheek biting*, has not been associated with malignant transformation. However, cheek/lip biting usually does not last indefinitely and could be easily modified if local (sharp teeth) and psychological situations change [12]. The* alveolar ridge keratosis *(ARK) locates in parts that are naturally prepared to endure mechanical irritation and could be considered as physiological adaptations of toothless alveolar ridges, similar to calluses on the skin. Consequently, ARK were not accounted as CMI in our study. Moreover, oral cancer is a multifactorial disease, and typically an accumulation of risk factors is needed. The primary aim of this study was to characterize CMI, and that is why other potential cofactors, such as chronic inflammation of various sources (immune diseases, bacterial or fungal infections, etc.), were not addressed.

Finally, this is a retrospective study, so an obvious limitation is that it may lack some information regarding alcohol and tobacco consumption. Therefore, a yes/no classification was used for those variables. Canine-protected articulation is widely accepted as a requisite for oral rehabilitation [30], and its absence was not recorded. In that situation, it is more likely to find unintentional mucosal biting, and the damage is increased if there is a sharp/rough tooth surface, or tongue interposition. In future studies about CMI, presence of canine-protected articulation should be properly recorded in order to avoid CMI underestimating.

## 5. Conclusions

- CMI associated lesions are produced by dental or prosthetic factors, typically in association with functional factors: among them, tongue interposition is the most common.
- CMI affects mainly tongue and buccal mucosa, although it could generate lesions in virtually any oral localization.
- In OC cases with tongue involvement, tongue interposition is the most frequent Functional Factor.
- CMI associated with CTU shows features of origin, localization, and time span that are similar to CMI in OC.

CMI should be properly recorded with as much attention and detail as alcohol and tobacco consumption, paying special attention to dental and prosthetic factors, and its potential interactions with functional factors as well.

## Figures and Tables

**Table 1:** Demographics features and tobacco and alcohol status.

Variable	Category	BIML	CTU	Oral Cancer	*p* value
Age (mean)		42.26 (12–89)	45.89 (18–73)	63.89 (40–85)	**<0.0001** ^a^
Gender	Female	127	32	17	**0.03** ^b^
	Male	76	12	21	
Tobacco	Yes	108	23	18	0.8^b^
	No	95	21	20	
Alcohol	Yes	128	27	25	0.91^b^
	No	75	17	13	

BIML, Benign Irritative Mechanical Lesions; CTU, Chronic Traumatic Ulcer; ^a^Kruskal-Wallis. ^b^Chi-square.

**Table 2:** Distribution of CMI factors in BIML, CTU, and OC groups.

Variable	Group			*χ* ^2^ *p* value
	BIML (*n* = 203)	CTU (*n* = 44)	OC (*n* = 38)	
*Dental factors*				
Malposition	82	17	20	0,336
Diastema	56	18	12	0,214
Sharp teeth	144	41	32	**0,003**
Sharp/rough restorations	92	18	17	0,867
*Prosthetic factors*				
Dentures	76	20	21	0,099
Sharp/rough dentures	56	9	17	0,042
Denture retainers	37	16	6	**0,019**
Overextended flanges	27	13	8	**0,024**
Lack of stability	69	20	21	**0,028**
Lack of retention	37	14	19	**0,000**
*Functional factors*				
Functional (total)	152	36	26	0,373
Tongue interposition	47	21	16	**0,001**
Sucking	56	9	9	0,584
Biting	121	26	16	0,130
Others	49	8	5	0,564

CMI, Chronic Mechanical Irritation; BIML, Benign Irritative Mechanical Lesions; CTU, Chronic Traumatic Ulcer; OC, Oral Cancer. Bold denotes statistical significance.

## Abbreviations

CMI: Chronic Mechanical Irritation
OC: Oral Cancer
CTU: Chronic Traumatic Ulcer
BIML: Benign Irritative Mechanical Lesions
DF: Dental factors
PF: Prosthetic factors
FF: Functional factors.

## References

[1] Regezi J. A., Sciubba J. J., Jordan R. C. K. (2012). *Oral Pathology: Clinical Pathologic Correlations*.

[2] Lanfranchi H. E. (2002). Estomatologia y PTR. *Prótesis Total Removible: Fundamentos, Técnicas y Clínica en Rehabilitación Bucal*.

[3] Grinspan D. (1970). *Enfermedades de la Boca: Semiología, Patología, Clínica y Terapéutica de la Mucosa Bucal (Volume II)*.

[4] Piemonte E. D., Lazos J. P., Brunotto M. (2010). Relationship between chronic trauma of the oral mucosa, oral potentially malignant disorders and oral cancer. *Journal of Oral Pathology and Medicine*.

[5] Thumfart W., Weidenbecher M., Waller G., Pesch H.-J. (1978). Chronic mechanical trauma in the aetiology of oro-pharyngeal carcinoma. *Journal of Maxillofacial Surgery*.

[6] Velly A. M., Franco E. L., Schlecht N. (1998). Relationship between dental factors and risk of upper aerodigestive tract cancer. *Oral Oncology*.

[7] Rosenquist K., Wennerberg J., Schildt E.-B., Bladström A., Göran Hansson B., Andersson G. (2005). Oral status, oral infections and some lifestyle factors as risk factors for oral and oropharyngeal squamous cell carcinoma. A population-based case-control study in southern Sweden. *Acta Oto-Laryngologica*.

[8] Pérez M. A., Raimondi A. R., Itoiz M. E. (2005). An experimental model to demonstrate the carcinogenic action of oral chronic traumatic ulcer. *Journal of Oral Pathology and Medicine*.

[9] Keibel A., Singh V., Sharma M. C. (2009). Inflammation, microenvironment, and the immune system in cancer progression. *Current Pharmaceutical Design*.

[10] Sato T. (1995). A study on effect of mechanical irritation in development and progression of tongue cancer. *Kobubyo Gakkai Zasshi*.

[11] Anura A. (2014). Traumatic oral mucosal lesions: a mini review and clinical update. *Oral Health and Dental Management*.

[12] Burket L. W., Greenberg M. S., Glick M., Ship J. A. (2008). *Burket's Oral Medicine*.

[13] Randhawa T., Shameena P. M., Sudha S., Nair R. G. (2008). Squamous cell carcinoma of tongue in a 19-year-old female. *Indian Journal of Cancer*.

[14] Kumar M., Pal Singh P., Saxena D., Singla N. (2014). Chronic trauma as precipitating factor of squamous cell carcinoma of tongue—3 case reports. *Indian Journal of Dental Sciences*.

[15] Huang J., He B., Chen F. (2015). Association between oral hygiene, chronic diseases, and oral squamous cell carcinoma. *Zhonghua Yu Fang Yi Xue Za Zhi*.

[16] Christopher A. F., Gupta M., Bansal P. (2016). Micronome revealed miR-19a/b as key regulator of SOCS3 during cancer related inflammation of oral squamous cell carcinoma. *Gene*.

[17] Khawaja S. N., Nickel J. C., Iwasaki L. R., Crow H. C., Gonzalez Y. (2015). Association between waking-state oral parafunctional behaviours and bio-psychosocial characteristics. *Journal of Oral Rehabilitation*.

[18] Lambrechts H., De Baets E., Fieuws S., Willems G. (2010). Lip and tongue pressure in orthodontic patients. *European Journal of Orthodontics*.

[19] Gupta B., Lalloo R., Johnson N. W. (2015). Life course models for upper aero-digestive tract cancer. *International Dental Journal*.

[20] LaPlante N. C., Singhal S., Maund J., Quiñonez C. (2015). Visits to physicians for oral health-related complaints in Ontario, Canada. *Canadian Journal of Public Health*.

[21] Gorsky M., Silverman S. (1984). Denture wearing and oral cancer. *The Journal of Prosthetic Dentistry*.

[22] Talamini R., Vaccarella S., Barbone F. (2000). Oral hygiene, dentition, sexual habits and risk of oral cancer. *British Journal of Cancer*.

[23] Vaccarezza G. F., Ferreira Antunes J. L., Michaluart-Júnior P. (2010). Recurrent sores by ill-fitting dentures and intra-oral squamous cell carcinoma in smokers. *Journal of Public Health Dentistry*.

[24] Rotundo L. D. B., Toporcov T. N., Biazevic G. H., de Carvalho M. B., Kowalski L. P., Antunes J. L. F. (2013). Are recurrent denture-related sores associated with the risk of oral cancer? A case control study. *Revista Brasileira de Epidemiologia*.

[25] Manoharan S., Nagaraja V., Eslick G. D. (2014). Ill-fitting dentures and oral cancer: a meta-analysis. *Oral Oncology*.

[26] Brady S. L., Wesling M. W., Donzelli J. J. (2016). Swallowing frequency: impact of accumulated oropharyngeal secretion levels and gustatory stimulation. *Ear, Nose & Throat Journal*.

[27] Lockhart P. B., Norris C. M., Pulliam C. (1998). Dental factors in the genesis of squamous cell carcinoma of the oral cavity. *Oral Oncology*.

[28] Rivera C. (2015). Essentials of oral cancer. *International Journal of Clinical and Experimental Pathology*.

[29] Sharan R. N., Mehrotra R., Choudhury Y., Asotra K. (2012). Association of Betel nut with carcinogenesis: revisit with a clinical perspective. *PLoS ONE*.

[30] El Kerdani T., Nimmo A. (2016). Integrating conventional and CAD/CAM digital techniques for establishing canine protected articulation: a clinical report. *Journal of Prosthetic Dentistry*.

